# A three‐goal model for patients with multimorbidity: A qualitative approach

**DOI:** 10.1111/hex.12647

**Published:** 2017-11-28

**Authors:** Neeltje P. Vermunt, Mirjam Harmsen, Glyn Elwyn, Gert P. Westert, Jako S. Burgers, Marcel G. Olde Rikkert, Marjan J. Faber

**Affiliations:** ^1^ Scientific Center for Quality of Healthcare (IQ healthcare) Radboud Institute for Health Sciences Radboud University Medical Center Nijmegen The Netherlands; ^2^ The Dutch Council for Health and Society (*Raad voor Volksgezondheid en Samenleving*, RVS) The Hague The Netherlands; ^3^ The Dartmouth Institute for Health Policy & Clinical Practice Lebanon NH USA; ^4^ Cochrane Institute for Primary Care and Public Health Cardiff University Cardiff UK; ^5^ Family Medicine Department School for Public Health and Primary Care (CAPHRI) Maastricht University Maastricht The Netherlands; ^6^ Dutch College of General Practitioners Utrecht The Netherlands; ^7^ Radboud University Medical Center/Radboudumc Alzheimer Center Nijmegen The Netherlands

**Keywords:** collaborative goal‐setting, decision making, elderly, fundamental goals, goal‐oriented care, multimorbidity

## Abstract

**Background:**

To meet the challenge of multimorbidity in decision making, a switch from a disease‐oriented to a goal‐oriented approach could be beneficial for patients and clinicians. More insight about the concept and the implementation of this approach in clinical practice is needed.

**Objective:**

This study aimed to develop conceptual descriptions of goal‐oriented care by examining the perspectives of general practitioners (GPs) and clinical geriatricians (CGs), and how the concept relates to collaborative communication and shared decision making with elderly patients with multimorbidity.

**Method:**

Qualitative interviews with GPs and CGs were conducted and analyzed using thematic analysis.

**Results:**

Clinicians distinguished disease‐ or symptom‐specific goals, functional goals and a new type of goals, which we labelled as fundamental goals. “Fundamental goals” are goals specifying patient's priorities in life, related to their values and core relationships. These fundamental goals can be considered implicitly or explicitly in decision making or can be ignored. Reasons to explicate goals are the potential mismatch between medical standards and patient preferences and the need to know individual patient values in case of multimorbidity, including the management in acute situations.

**Conclusion:**

Based on the perspectives of clinicians, we expanded the concept of goal‐oriented care by identifying a three‐level goal hierarchy. This model could facilitate collaborative goal‐setting for patients with multiple long‐term conditions in clinical practice. Future research is needed to refine and validate this model and to provide specific guidance for medical training and practice.

## 1 INTRODUCTION

Interest in goal‐oriented care is increasing among policy makers and clinicians alike.^1^, ^2^, ^3^, ^4^, ^5^, ^6^, ^7^ Goal‐oriented care is particularly important where patients have multiple long‐term conditions, known as multimorbidity. Multimorbidity is defined as the coexistence of two or more chronic diseases or conditions, and its incidence is causing a challenge to health‐care systems, patients and medical practitioners. In daily medical practice, multimorbidity challenges decision making in several ways. Disease priorities can be interfering^8^, ^9^ and the need of adherence to multiple disease guidelines can be problematic.^10^ Disease‐specific guidelines are often not applicable to older patients with multiple conditions^7^, ^9^, ^11^, ^12^ and compliance to multiple single disease guidelines can result in polypharmacy, high treatment burden, inattention to social and personal context and failure to align care with personal goals and preferences.^13^, ^14^ Having multiple chronic conditions often leads to the involvement of several clinicians, who concentrate on managing different conditions and monitoring different disease‐specific outcomes. Patients are at risk of receiving fragmented care that might lack focus on what matters most to them.^7^ Focusing care on what matters most to patients could be helpful but is also a challenge in itself.

Aligning health outcomes with individuals’ values is complex, especially for older adults with multiple chronic morbidities facing conditions with clinical uncertainty (eg cancer).^15^, ^16^, ^17^ Clinicians are challenged in helping people prioritize their values, define treatment goals and frame preferences in ways that are clinically relevant and aligned with one's values when faced with multiple diagnostic and treatment options.^18^, ^19^ Patients and clinicians may also differ in perspectives and priorities in this respect.^6^, ^9^, ^16^, ^19^, ^20^, ^21^, ^22^ Clinicians are at risk to make inaccurate assumptions about patient values and preferences^3^ and may think that they know what is best for patients.^10^ It is clear that in care for older patients with multimorbidity, incorporating values and preferences in decision making is necessary to focus on what matters most to them, but in daily practice this is complex.

To meet the challenges of multimorbidity care, including the necessary incorporation of values and preferences, Tinetti et al^2^ proposed a shift from a disease‐oriented to a goal‐oriented approach. Taking this approach, it seems health goals can be defined using a range of dimensions (eg symptoms; physical functional status, including mobility; and social role). In goal‐oriented health care, care is personalized to accommodate patients’ goals, preferences and resources.^3^, ^23^ Collaborative goal‐setting (CGS), defined as “a process by which health‐care professionals and patients agree on a health‐related goal”,^15^ can be useful for personalizing care and encourages patient involvement in the goal‐setting process. CGS has been evaluated in several rehabilitation settings.^24^, ^25^, ^26^, ^27^ However, CGS in the context of older patients with multimorbidity is not common practice yet. In the 2014 Commonwealth Fund Survey of adults aged 65 or older and having a chronic condition, rates of respondents reporting the sharing goals with a professional varied from 23% (Sweden), till 59% (United Kingdom). Nine of 11 countries were having rates of less than 50% of respondents reporting the sharing goals with a professional.^28^ There appears to be a relative lack of insight in goal‐setting processes in the presence of complexity and little evidence to support best practices in goal‐setting with complex patients.^29^ Furthermore, as concluded by Knight et al, the concepts of values, goals and preferences are often used interchangeably,^30^ indicating a need for establishing consistent definitions. In the Netherlands, the current views of general practitioners (GPs) and clinical geriatricians (CGs) on the concepts of goals and CGS are as yet unknown. These views could provide valuable input into the concept of goal‐orientation and into the perceived relevance of the approach in clinical practice. The objective of this study was to examine the concept of goal‐orientation from a clinician's perspective, in the context of CGS and shared decision making (SDM), where patients have multiple long‐term conditions.

## 2 METHOD

### 2.1 Participants

This qualitative study was conducted by inviting CGs and GPs to participate in semi‐structured interviews. Participants were selected using a purposive and snowball method, aiming to recruit professional experts, and contacted by email. We invited experienced GPs and CGs performing research, teaching, developing or implementing specific innovations in care for older patients. The first two participants were acquaintances of the interviewer (first author). Some GPs were recruited at a meeting of GPs holding a specialization in geriatric care. In the sampling, we aimed to recruit comparable numbers of CGs and GPs. To obtain diverse perspectives, we tried to ensure that different types of practice and practice location (rural or urban) for GPs and different types of hospitals (CGs) would be represented. Furthermore, we tried to ensure that all Dutch regions would be represented as much as possible. The response rates of CGs and GPs approached were 86% and 54%, respectively. The final sample consisted of 18 CGs and 15 GPs.

### 2.2 Procedures

An interview guide (Table 1) was inspired by two perspectives on goal‐oriented health care for elderly patients with chronic multimorbidity^2^, ^3^ and professional experiences (general practice and clinical geriatrics) in our research team. Two pilot interviews were conducted with a CG and a GP. Main topics and subtopics were not changed based on the pilot interviews nor during the conducting of the interviews. The interview guide covered three main topics: CGS, SDM and effective collaborative action. We defined *effective collaborative action* as clinicians and patient deciding on and performing diagnostic and treatment steps in line with collaborative goals, which were set between patient and clinicians or with other involved caretakers. Definitions were not given to the interviewees. At the start of the interview, the clinicians were asked to use the context of regular care for community‐dwelling older patients (age >75 years) with a chronic disease or multimorbidity without further specifications. It was also suggested to keep one or more cases in mind in answering the questions. All topics and subtopics were covered in all interviews. Interviews could differ in asking further questions for a better understanding of an interviewee's answer.

**Table 1:** Main topics of the semi‐structured interview guide

Main topics	Subtopics per main topic
Introduction of the interview	
Collaborative goal‐setting between medical practitioners and patients	Definition of the conceptExperiences and process descriptionTypes of goalsBarriers and facilitators
Collaborative goal‐setting within a collaborative framework of multiple medical practitioners	Experiences and expectationsRolesBarriers and facilitators
Shared decision making between medical practitioners and patients	Definition of the conceptExperiences and expectationsBarriers and facilitators
Shared decision making within a collaborative framework of multiple medical practitioners	Experiences and expectationsRolesBarriers and facilitators
Effective collaborative action between multiple medical practitioners	Definition of the conceptExperiences and expectationsRolesBarriers and facilitators
Relationships between the examined concepts of collaborative goal‐setting, shared decision making and effective collaborative action	Relationships between the conceptsDesirability of these processesPossible actions to stimulate
Conclusion of the interview	Conclusion of the interview

The first author, who is trained as a GP, conducted the interviews between November 2012 and April 2013. The interview duration was approximately 60 minutes and they were conducted face‐to‐face or by telephone. All interviews were audio‐recorded and transcribed. Detailed field notes were made after each interview. Theoretical memos were drafted throughout the data collection and analysis process. The two final interviews confirmed theoretical saturation as they did not reveal new issues or topics.

### 2.3 Analyses

Inductive thematic analysis was used for analysis.^31^ Thematic analysis is an approach for qualitative research focusing on identifying, analyzing and reporting patterns (themes) within qualitative data and the interpretation of aspects of the research topic. In an inductive approach, themes are data‐driven. An iterative process of interviewing and analysis was followed. During the interviewing phase, preliminary analyses were conducted based on reflections and discussion of the interviews (first and last author) and by constantly comparing the interviews with the field notes. These preliminary analyses were conceived in theoretical memos, and the interview guide was continually adapted to reflect emerging insights.

In the coding process, data were conceptually interpreted and labelled accordingly. The two data coders (first and second author) applied open coding to the first five transcripts. Initial codes were compared, discussed, grouped and categorized to develop an initial coding tree. The first five interviews were coded independently by both data coders. The remaining interviews were coded by one researcher (second author) and checked by the other (first author). In weekly meetings, the researchers (first and second author) compared, discussed and agreed on the coding of the transcripts, including the creation of additional codes and further refinement of categories and subcategories. Similarities, differences, regularities and patterns were interpreted and discussed to identify themes and to generate hypotheses. Illustrative quotations were selected to underpin and illustrate our findings. In addition, Box 1 presents two case examples, one of a GP and one of a CG to illustrate daily practice of this topic.

Box 1 **Two Case Examples from Daily Practice**

**Case Example One**

GP_10 spoke about a patient of over 90 years old whose hip surgery had failed.

[The prosthesis] got infected, so her hip had to be removed (…) She was admitted to a nursing home (…), but she really wanted to return home. I understood why, because she had an unusual background. She had been interned in a concentration camp years before that (…). All she really wanted was to go home, because that was the only place she felt safe (…). Everything around her reminded her of her traumatic experiences (…). She actually returned to her apartment in that severely disabled state. But she coordinated all her care and assistance there (…) and lived for years, in fact. Naturally, this is an extreme case, but if you look at the patient's circumstances and history, it is completely understandable (…) and her final years were wonderful. Yes, they were.

**Case Example Two**

CG_11 spoke about a patient who was referred by the GP because of abdominal pains, whereas this patient had been screened by the internist 3 years earlier revealing no major diseases.

The GP still was not sure: Couldn't there still be a malignancy, isn't there anything else still? He did not have a conversation with the patient asking: “If we refer you to that hospital now, what would be your goal? And what is your goal in life in general?” (…) I came to an agreement with (…) the patient: “OK, we are going to do some examinations” (…), but we also immediately talked about: “What would you actually want?” And then she said: “I really just want the abdominal pains to go away” (…) She was very clear about her concerns: “It is not my main concern whether there is a malignancy or not.” (…)Then you talk it over in a conversation with the patient. If you've set that goal for yourself: “Now, how far do we want to go to see if we can help you get there?” And together you decide that, at this moment, a colonoscopy and a gastroscopy are really too much for the patient. And yes, a patient then accepts that certain issues cannot be completely figured out. But we do as much as we can to help her achieve her goals.

*Note:* These are two case examples from daily practice that show the importance of aligning care with patients’ personal history, values and priorities and its difficulties.

The quotations were translated from Dutch into English by a professional translator. The translator and first author discussed the translations to ensure that proper meaning of words and nuances were kept in the translation process. For data coding and analysis, Atlas‐ti 7.1.15, (GmbH, Berlin, Germany), was used.

### 2.4 Quality assurance

The Consolidated Criteria for Reporting Qualitative Research (COREQ) and a 15‐point checklist for thematic analysis by Braun and Clarke were used for design, performance and reporting.^31^, ^32^ Appendix S1 reports on these COREQ criteria in relation to our research. All interview topics were analyzed in one process to secure consistency and theoretical interrelatedness.

## 3 RESULTS

Participating GPs’ (n = 15) mean age was 51 years, being 40% male and on average having 16 years of professional experience. Participating CGs (n = 18) had a mean age of 48 years, being 50% male, and having on average 10 years of professional experience. Further participants’ characteristics are presented in Table 2. Three themes were identified (Box 2).

**Table 2:** Basic characteristics of participants

Characteristics	General practitionern = 15	Clinical geriatriciann = 18
Age, M (SD) (years)	51 (6.6)	48 (8.6)
Gender, n (% men)	6 (40)	9 (50)
Practice type, n (%)		N/A
Single	1 (7)	
Duo	2 (13)	
Group/Health Centre	12 (80)	
Physician assistant in geriatric care^a^, n (% yes)	12 (80)	N/A
Type of Hospital, n (%)	N/A	
Academic Centre		3 (17)
Community Hospital		9 (50)
Mental Care Facility		2 (11)
Non‐Academic Teaching Hospital		4 (22)
Researcher, n (% yes)	5 (33)	9 (50)
Supervisor, n (% yes)	3 (20)	11 (61)
GP specialized in Geriatric Care, n (% yes)	9 (60)	N/A
Years of Professional Experience, median (range)	16 (3‐34)	10 (3‐22)

N/A, not applicable; M, mean; SD, standard deviation; GP, general practitioner.

a In GP practice.

Box 2 **Themes**

1

1. Clinicians draw distinctions between different types of goals, namely disease‐specific or symptom‐specific goals, functional goals, and fundamental goals.
2. The consideration of fundamental goals.
3. The relevance of explicit goals for decision making.

### 3.1 Clinicians draw distinctions between different types of goals

From the data, three types of goals were identified, that is disease‐specific or symptom‐specific goals, functional goals and a third category labelled as fundamental goals.

*Fundamental goals* were described as goals specifying a patient's priorities in life, such as their values and core relationships, topics**,** that serve as reference points for decision making. These are goals considering the patient's personal views on what constitutes quality of life. Fundamental goals concern questions such as: “What makes your life worth living?”; “How do you lead your life?”; “What are your views on end of life?”; “How do you feel about quality of life vs lengthening of life?”. Examples provided by medical practitioners include: “being of help to others and/or society”, and “no wish for changes”. “Being able to continue living independently” is a goal often mentioned by patients, according to medical practitioners, for example CG_29:

Almost invariably, the main goal for this target group is to continue living independently (…) And that ability to continue living independently is frequently more important to [patients] than being treated in a residential care facility or nursing home. (CG_29)

Fundamental goals reflect a patient's view on their own future in the broadest sense.

*Functional goals* were described as goals related to reducing limitations in functioning. Examples include “being able to wash or dress oneself”, “driving a car, and “staying mobile”. GP_06 described functional goals as follows:

What is important and what should we focus on? (….) Is it an issue if people are only able to go to a convenience store or supermarket? Or is the problem that they can no longer shop or dress independently? (GP_06)

*Disease‐specific* or *symptom‐specific goals* are goals relating to the diagnosis or treatment of a specific disease or symptom. Patients may ask for example for the reduction in distress caused by symptoms like shortening of breath, itching or pain. In a goal‐setting process, clinician and patient can set a patient symptom‐specific goal together, which incorporates personal choices in diagnostic trajectories and treatments. Some patients, for example, do not want to engage in all kinds of diagnostic trajectories as long as a certain symptom can be reduced by a certain symptomatic treatment. Other patients want to know what is causing the symptom. This type of goals can also originate from a certain disease. An example is a patient asking for disease‐specific medication, as mentioned by CG_21:

These goals vary largely per patient. They can be very explicitly related to the disease. Conceivably, for instance, a patient may make a very specific request for “a pill against dementia”. (CG_21)

### 3.2 The consideration of fundamental goals

The practitioners differed in their consideration of fundamental goals, creating three orientation categories, that is (i) no consideration of fundamental goals, (ii) implicit consideration of fundamental goals and (iii) explicit consideration of fundamental goals.

#### 3.2.1 No consideration of fundamental goals

Practitioners in this category mentioned a primary focus on functional goals and/or disease‐specific or symptom‐specific goals. Functional goals and disease‐specific or symptom‐specific goals can be connected to each other as described by CG_12:

Those [patients] usually come to me with problems (…). Their complaints vary from “more trouble walking” to “tiring out faster”, “forgetfulness”, “falling” and a whole range of other problems. You try to unravel all their problems and often come back to their medical diagnosis. At that point, you try to figure out how you can help. But the foundation is still the patient's functioning (…). (CG_12)

The practitioners in this category did not mention setting or taking into account fundamental goals.

#### 3.2.2 Implicit consideration of fundamental goals

Practitioners in this category were aware of fundamental goals. However, these goals were presumed but not made explicit in a discussion with the patient. GP_04 illustrates that they are aware of implicit fundamental goals while focusing on quality of life:

At present, what I really find important is that we mainly focus on the quality of life of the elderly and take into account their opinions and preferences. In terms of actual practice, I cannot say that my colleague and I have specific discussions [with our patients] on a regular basis about the goals patients want to pursue in their life. However, based on the questions asked, we do pay attention to what is feasible for patients. We are also cautious when it comes to adding any new medicine, having in mind the issue of multiple medications and their side effects. It is a matter of weighing up everything very carefully. When it comes to elderly patients, it is important to figure out whether all interventions will benefit them. (GP_04)

#### 3.2.3 Explicit consideration of fundamental goals

The third category constitutes practitioners who have an orientation towards disease‐specific and/or functional goals, while explicitly taking fundamental goals into account. If fundamental goals are discussed and made explicit, other goals can be set in accordance with these fundamental goals, as illustrated by the following example from daily practice of the importance of quality of life as a reference point in decision making. What quality of life means to a specific patient, can only be assessed by that specific patient:

Now imagine I discover that someone has cancer and maybe I can still help them (…) in a way that allows the patient to live a few months longer. But then, of course, there is still the decision whether or not to treat him (…). Or do you choose limited treatment? That is something that must be agreed upon with specialists, the patient and, of course, with me (…) The core issue remains the quality of life. And (…) of course that is something I can assess, at least to a certain extent, but this will primarily be done by the patient. (GP_10)

This theme “The consideration of fundamental goals” makes clear that although aspects of implicit fundamental goals may be taken into account, discussion and consideration of aspects of explicit fundamental goals, are not regular practice yet. Table 3 provides several quotations of questions asked to elicit fundamental goals, as mentioned by the practitioners. These practitioners’ examples were transformed by the authors into possible questions, which may be helpful to use in clinical practice to start a discussion on fundamental goals.

**Table 3:** Example questions for collaborative fundamental goal‐setting

Example questions	Quotations
How do you see your future? How would you prefer to plan it?	GP_15: I mean, you have to consider how these individuals see their future (…) and how they prefer to shape that future….
Where are you from and to what extent does spirituality play a role in your life? How do feel about the different aspects of your life? How do you envision the end of your life?	GP_21: On the one hand, I ask everyone over 75 about their core values and quality‐of‐life values. As for the extent of their spiritual experiences, and where they come from (…), we are not in a position to deal with that (…). Regarding quality‐of‐life values, those tend to relate to things like whose children visit first or (…) whether the garden is still blooming, etc. (…) Based on the core values and quality of life and other [things], we can retrieve a clear picture. At any rate, there is a lot of similarity. Is advance care planning more of a medical process? (….) If so, how do you start your daily life and how exactly do you end it? (….) When do you want that to happen? (….) This is what the patients’ vision of the end of their life entails (…) in terms of core values and quality‐of‐life values
What is important to you? What do you want and what do you want to avoid? What do you consider important? What are you afraid of?	CG_17: “What do I find important?”; “What do you really want and what do you want to avoid?” (…) “What do you consider important?”; “What are you afraid of?”
What are your goals and what do you want from life, specifically?	GP_10: “What are your goals and what do you want from life, specifically?” This question is obviously very essential. The first things that come to mind, of course, are end‐of‐life decisions, such as entering a nursing home, continue living independently, undergoing euthanasia or refusing it. That period, however, is just one aspect, and it comes at the very end. Before that point, there is so much more: decisions about how to live and whether or not to accept medical treatment. So the decision‐making process concerns treatment, referral, end of life and place of residence

The example questions are based on illustrative examples given by the interviewees.

### 3.3 The relevance of explicit goals for decision making

The analysis revealed several reasons to explicate fundamental goals. The patient's preferences are not always in line with medical standards, nor with the preferences of the practitioners involved, as is illustrated by CG_03:

I really do believe that care will become better for the patient, that they will finally get the care they want instead of the care that guidelines, or we together, say they must be given, whereas that is not what they want. (CG_03)

Secondly, patients’ preferences may vary. For example, CG_17 describes differences in medication preferences in a case of dementia:

Some people are keen to try this medication which might improve their memory (…) But there are also people who say: “If it leads to weight loss or gives me skin problems, then I don't want it”. (CG_17)

In the event of multimorbidity, the consideration of goals is even more important. The more complicated a patient's situation, the more important it is to incorporate what constitutes quality of life to a patient in decision making as described by GP_28:

The more complicated the situation, that is the more medical issues someone is suffering from, the more the focus lies on quality of life and on the interaction between various conditions and what that means to someone (…). Then, it becomes more important to know what patients want for themselves, as it is important to have ideas about that (…). So basically, the larger the extent of multimorbidity, the more important it is to know what is important to the patient. (GP_28)

A discussion of fundamental goals can be helpful to make this meaning of quality of life, what people want for themselves in a broader sense and what is important to someone, explicit. Finally, discussing fundamental goals explicitly can provide important information for acute situations that may occur in the future, as is emphasized by CG_14:

When it comes to vulnerable elderly people, the circumstances that require you to make important decisions often arise unexpectedly (…). This may happen, for instance, when their regular doctor is absent and a different doctor is on duty (…) or by an emergency doctor in the hospital (…) When these situations occur, it is really helpful to be able to rely on information you have exchanged earlier on. (CG_14)

## 4 DISCUSSION

### 4.1 Main findings

The case examples (Box 1) demonstrate the importance and difficulties of aligning care with patients’ personal histories, values and priorities. Our analysis revealed three types of goals: disease‐specific or symptom‐specific goals, functional goals and a third type of goals, which we labelled fundamental goals. From our analysis followed that fundamental goals are implicitly and explicitly applied in daily practice. We hypothesize that the explicit setting and application of fundamental goals could lead to patient‐specific clinical decisions concerning diagnostic trajectories or treatments by translating values, personal history and core relationships into useable reference points for decision making.

### 4.2 Interpretation

Earlier studies confirmed our findings. Maintaining (functional) independence, fixing specific symptoms or functional challenges, day‐to‐day functioning, behaviour and emotional health and safety are considered important goals and priorities.^33^, ^34^ An analysis of health‐related values of multimorbid cancer survivors revealed the five values: self‐sufficiency, life enjoyment, connectedness and legacy, balancing quality and length of life, and engagement of care.^18^ Incorporating patient values into health‐care decisions is critical, especially for elderly patients since goals may change when life expectancy shortens.^35^ However, there appears to be a lack of consistency in the use of the concepts of values, goals and preferences.^18^, ^30^ Naik et al^18^ make a distinction between values and health‐care goals, whereby goals and preferences are seen as more context or circumstance specific. Values usually are stable and can be seen as fundamental beliefs about one's self and life. Our findings are consistent with the importance and guiding role of values, as stated by Naik et al.^18^ However, although most people would intuitively agree with the importance of incorporating values in decision making, this seems to be not an easy ‘job’. Insight in approaches to actually clarify values and elicit patient preferences in a structured and consistent manner is lacking.^36^

**Figure 1:**
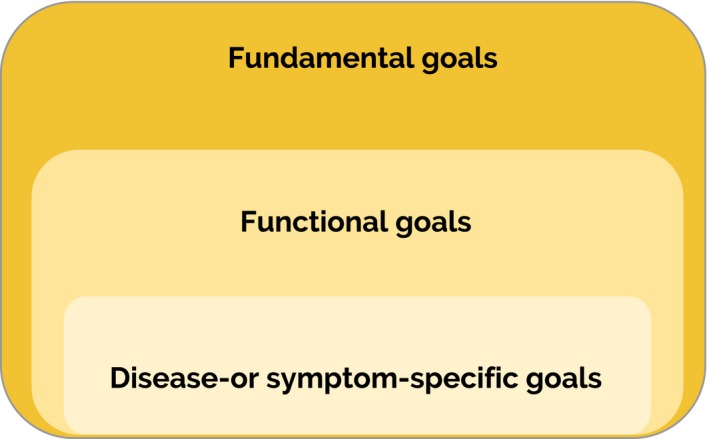
Three‐goal model for clinical practice. This figure shows that disease‐specific or symptom‐specific goals flow from functional goals and both flow from fundamental goals

As a synthesis of the three themes identified from the data, Figure 1 represents a three‐goal model for clinical practice showing three levels of relevant goals in caring for elderly with multimorbidity. Within this three‐goal model, types of goals are interrelated, with disease‐specific or symptom‐specific goals flowing from functional goals, and both flowing from fundamental goals. Symptom‐specific goals, for example, incorporate personal choices in diagnostic trajectories and treatments. These personal choices are based on aspects like beliefs, personal history, core relationships, values and functioning. Explicit fundamental and functional goals represent these aspects and are thereby useful in the related goal‐setting process of symptom‐ or disease‐specific goals.

Fundamental goals can be seen as a translation of elements like values, core relationships and priorities in life, into concrete goals. If, for example, both autonomy (quality of life at home above prolonging life in a nursing home) and staying with and taking care of your disabled child as long as possible, are important to a person, this conflict and/or trade off in these important values/life goals/core relationships can be discussed and translated into a fundamental goal. For example, a fundamental goal could be to prolong life as long as possible, even with the chance of having to stay in a nursing home, provided that this person remains cognitively able to self‐manage his or her life and that of his or her disabled child. In this sense, fundamental goals translate rather abstract elements such as values, acknowledging nuances and trade‐offs in a certain context. Explicit awareness of all three goal levels and their interrelatedness is needed, although the emphasis on a certain type of goal in a specific care situation will be dependent on patient‐specific, professional‐specific and contextual factors. This three‐goal model may provide a guide for CGS and the consideration of explicit goals in decision making with patients with multiple long‐term conditions.

The three‐goal model could be relevant for individualized management or care plans. In case of multimorbidity, a dynamic individualized care plan is recommended.^9^, ^37^, ^38^, ^39^, ^40^ Core elements of these plans are “optimizing quality of life, eliciting preferences and goals, weighing risks and benefits of implementing recommendations from single disease guidelines, addressing trade‐offs, setting priorities, stopping potentially harmful or unnecessary medications and starting beneficial medications while simplifying regimens, integrating care, and minimizing treatment burden”.^37^ In individualized care plans, values are seen as guiding principles. Using the three‐goal model in individualized care plans may be helpful to actually use values as guiding principles. In a process of fundamental goal‐setting, values are translated into explicit fundamental goals, thereby also incorporating elements as personal history and core relationships. These explicit fundamental goals can be used as input for the elicitation of the other goal types. In this way, a goal‐setting approach of different types of interrelated goals actually incorporates values into care plans and health‐care decisions.

Berntsen et al^40^ developed a goal typology with a distinction between professionally defined and personally defined goals. Personally defined goals are goals which “honor the patient's right to make decisions about his/her personal matters”. Personal goals “amount to a personal construction of what ‘health’ means for the individual”.^36^ These personal goals are used to justify the choice of the goals a professional should pursue. Our three‐goal model differs from Berntsen's framework in two ways. Considering content, fundamental goals are based on and include values, aspects of personal history, individual priorities in quality of life and core relationships, thereby constituting a further elaboration on the concept of personal goals. Furthermore, in contrast to Berntsen et al, all types of goals are basically elicited jointly, although the weight of the patient's and the professional's input may vary for different types of goals. Although fundamental goals are very personal and can be difficult to construct, elicit and share, discussing and explicating these goals is a collaborative process between patient and practitioner. In our model, all types of goals are joint goals in this sense and not exclusively patient or professional goals.

It must be noted that fundamental goals and CGS show similarities with advance care planning (ACP). ACP is a formal decision‐making process that aims to support patients in making decisions about future care in anticipation of the incapacity to make decisions due to a worsening condition.^41^ Patients consider the focus in health care on patient goals and values to be particularly helpful.^42^ ACP is usually part of an end‐of‐life care strategy and is used in the context of progressive illness and anticipated deterioration.^43^ In our view, discussing and explicating a patient's fundamental goals and specifying values and underlying beliefs and preferences, could also be valuable in earlier stages of life, especially in patients with multimorbidity.

### 4.3 Strengths and limitations

The methodological strengths of this study include the following: First, we worked with an interviewer who is trained as a GP, which may have encouraged the participants to speak frankly and directly from their own professional perspectives. The second coder has substantial experience in interview analysis but has no medical background, which helped us avoid a ‘medical’ bias in our data interpretation. Second, a purposive sampling and snowball extension method was used to recruit professional experts. In the Netherlands, both GPs and CGs deliver medical care to elderly people living independently, but they provide care in different settings. In this phase of theory development, we consider GPs and CGs to be complementary, as they both contribute to the saturation of data collection on current medical thinking on these themes. We are aware that our purposive sampling and snowball extension participant selection method has a risk of bias in the sense that the results cannot be generalized to the whole Dutch GP and CG population. However, although our selection of participants is not representative for the whole GP and CG population, these are representatives who can be considered specifically interested and busy in developing clinical practice, especially care for older patients, further. We considered recruitment of these professional experts a necessity to answer our research question, because a goal‐oriented approach and CGS more specifically are not yet broadly implemented. Considering participants’ basic characteristics (Table 2); these show considerable variability and comparability in line with the actual Dutch context, for example in case of practice type.

A limitation of the study is that the categories in the theme “The consideration of fundamental goals” were based on data analysis of the answers spontaneously given by the clinicians. The clinicians in the first category, “No consideration of fundamental goals”, explicitly mentioned a primary focus on functional goals and/or disease‐specific or symptom‐specific goals. They did not mention setting nor taking the type of goals, we eventually labelled as fundamental goals into account. Based on our results, we cannot be sure that they never take fundamental goals into account in their daily patient care; however, we can conclude that fundamental goals were not their primary point of orientation, otherwise they would have mentioned (aspects of) fundamental goals. We did not ask this during the interview, because this would potentially influence the results.

A further limitation of the study is that the model was developed on the basis of practitioners’ perspectives. Evaluation and adaptation of the model on the basis of an analysis of patients’ and caregivers’ perspectives is a high priority area. In addition, the impact of eliciting fundamental goals on the quality of decision making and of care requires future research.

### 4.4 Implications for practice and research

Further research on the patients’ perspectives on goals is required. Further combined theoretical and practice‐based research on this topic of goal‐orientation in the context of goal‐setting and decision making could prepare a shift in clinical practice towards goal‐oriented care for patients with multimorbidity.

## 5 CONCLUSION

This qualitative study provides new insights into types of goals and the consideration of goals in care for elderly patients with multimorbidity. Based on the perspectives of clinicians, we expanded the concept of goal‐oriented care by identifying a three‐level goal hierarchy acting as a guide to clinical care of patients with multiple long‐term conditions. Awareness of and application of explicit fundamental goals in addition to functional and symptom‐specific and/or disease‐specific goals could contribute in making daily care more patient goal‐oriented. Future research is needed to refine and validate the developed three‐goal model and to provide recommendations for medical training and practice.

## CONFLICT OF INTERESTS

Dr. Elwyn reports personal fees from Emmi Solutions, LLC, personal fees from Washington State Health Department, personal fees from National Quality Forum, personal fees from Radcliffe Press, personal fees from Oxford University Press, grants from Gordon and Betty Moore Foundation, during the conduct of the study; and Glyn Elwyn has initiated and led the Option Grid TM patient decision aids Collaborative, which produces and publishes patient knowledge tools in the form of comparison tables (http://optiongrid.org/). He has been a member of teams that have developed measures of SDM and care integration. These tools and measures are published and are available for use. For further information see http://www.glynelwyn.com/. The other authors have completed the ICMJE uniform disclosure form at www.icmje.org/coi_disclosure.pdf and declare the following: “For the submitted work, no support was received from any organization.” Neeltje Vermunt received financial support from her employer, the Council of Health and Society, as described under funding. No other relationships or activities have influenced the submitted work.

## APPENDIX S1

### Consolidated Criteria for Reporting Qualitative Studies (COREQ)

**Table:**

No	Item	Answer
Domain 1: Research Team and Reflexivity		
Personal Characteristics		
1.	Interviewer/facilitator	First author
2.	Credentials	MD, MSc
3.	Occupation	Senior advisor at an advisory council on public health and healthcare (public service) and PhD student.
4.	Gender	Female
5.	Experience and training	Economist and former general practitioner. PhD student. Courses on Atlas‐ti and qualitative research.
Relationship with participants		
6.	Relationship established	The sampling of potential participants was initiated by interviewing a GP and CG, who were both acquaintances of the interviewer; the other participants were not.
7.	Participant knowledge of the interviewer	Former general practitioner. Interviews belonging to PhD research.
8.	Interviewer characteristics	She has a background as a GP and an affinity for geriatric care. Considering working with an interviewer who is trained as a GP may have encouraged the participants to speak frankly and directly from their own professional perspectives. The second coder has substantial experience in interview analysis but has no medical background, which helped us avoid a ‘medical’ bias in our data interpretation.
Domain 2: Study Design		
Theoretical Framework		
9.	Methodological orientation and Theory	Inductive thematic analysis
Participant Selection		
10.	Sampling	A purposive and snowball method aiming to recruit professional pioneers.
11.	Method of approach	By email. Part of the recruitment of GPs took place at a broader meeting of GPs holding a specialization in geriatric care.
12.	Sample size	33 (18 clinical geriatricians and 15 general practitioners)
13.	Non‐participation	The response rates of clinical geriatricians and general practitioners were 86% and 54% respectively. Of the 21 CGs approached, one CG refused and two CGs did not respond to the first and reminder emails. A total of 28 GPs were approached. There were 6 non‐responders. 3 GPs responded positively, but did not respond to proposed dates. There were two drop‐outs (the interview was cancelled and there was no rescheduling (i.e. no response to proposed dates)). Two GPs of the same practice chose one participant. Their lack of time was the main reason not to participate.
Setting		
14.	Setting of data collection	Five interviews were face‐to‐face, the others were held by telephone, as the medical practitioners’ busy schedules and varying locations required flexibility. The face‐to‐face interviews were held at the interviewee's office.
15.	Presence of non‐participants	No
16.	Description of sample	See Table 2. Basic characteristics of participants
Data Collection		
17.	Interview guide	Main topics and subtopics are provided in table 1. A more detailed interview guide (in Dutch), including the introduction and closing of the interview, questions on the topics and questions on basic characteristics, is available upon request. In one case, the interview guide was sent to the interviewee in advance of the interview.
18.	Repeat interviews	No
19.	Audio/visual recording	Audio‐recording of all interviews
20.	Field notes	Yes
21.	Duration	Approximately 1 hour
22.	Data saturation	Yes
23.	Transcripts returned	3 interviewees wanted to receive the transcripts, which they did.
Domain 3: Analysis and Findings		
Data analysis		
24.	Number of data coders	2
25.	Description of the coding tree	Available upon request
26.	Derivation of themes	Derived from the data
27.	Software	Atlas‐ti 7.1.15
28.	Participant checking	Two participants provided feedback upon request.
Reporting		
29.	Quotations presented	Yes
30.	Data and findings consistent	Yes
31.	Clarity of major themes	Yes
32.	Clarity of minor themes	Yes
